# Os alimentos da Cesta Básica dos brasileiros são seguros para o consumo?

**DOI:** 10.1590/0102-311XPT182325

**Published:** 2026-03-23

**Authors:** Marcia Orth Ripke, Vanessa da Silva Corralo, Junir Antônio Lutinski

**Affiliations:** 1 Programa de Pós-graduação em Ciências da Saúde, Universidade Comunitária da Região de Chapecó, Chapecó, Brasil.

**Keywords:** Culturas Agrícolas, Limite Máximo de Agrotóxico em Alimentos, Inocuidade dos Alimentos, Agricultural Crops, Agro Toxic Maximum Allowable Limit on Food, Food Safety, Culturas Agrícolas, Límite Máximo de Agrotóxico en Alimentos, Inocuidad de los Alimentos

## Abstract

Objetivou-se analisar a presença de resíduos de agrotóxicos em alimentos da Cesta Básica dos brasileiros. Amostras de açúcar refinado, arroz polido longo fino tipo 1, café em pó tradicional, farinha de trigo branca tipo 1, feijão preto comum tipo 1 e óleo de soja refinado foram coletadas em supermercados no Mato Grosso, Paraná, Rio Grande do Sul e Santa Catarina. Os estados fazem parte da Região 3 de estratificação do Departamento Intersindical de Estatística e Estudos Socioeconômicos da Cesta Básica nacional. Os ingredientes ativos analisados foram os dez de maior volume comercializados para cada cultura, de 2019 a 2023, informados pelos departamentos de agricultura dos estados. Foram utilizadas estatísticas descritivas de frequência para caracterizar o estudo. Em amostras de arroz foram detectados resíduos de imazapir (Mato Grosso e Paraná) e triciclazol (Mato Grosso, Rio Grande do Sul e Santa Catarina). No café, o glifosato foi detectado no Paraná e em Mato Grosso e na farinha do Paraná, enquanto o glufosinato foi detectado na farinha de Santa Catarina. Em relação ao feijão, o glufosinato foi detectado em todos os estados, além do glifosato (Rio Grande do Sul) e carbendazim em Mato Grosso. Embora os resíduos de agrotóxicos tenham permanecido dentro dos limites legais, é preciso destacar que esses limites não garantem a ausência de risco. Exposições crônicas, especialmente em populações vulneráveis, e a soma de fontes alimentares diversas podem aumentar o potencial de efeitos negativos à saúde. Estratégias de redução da exposição, como optar por produtos de baixo risco ou orgânicos e aplicar técnicas complementares de mitigação, continuam sendo importantes.

## Abstract (en)

The aim of the present study was to analyze the presence of pesticide residues in foods of the Brazilian “staple food basket”. Samples of refined sugar, type 1 fine long polished rice, traditional powdered coffee, type 1 white wheat flour, type 1 common black beans, and refined soybean oil were collected from supermarkets in the states of Mato Grosso, Paraná, Rio Grande do Sul, and Santa Catarina. These states are part of Region 3 of the Inter-Union Department of Statistics and Socioeconomic Studies on the national staple food basket. The active ingredients analyzed were the ten with the highest volume sold for each crop from 2019 to 2023, as reported by the departments of agriculture of the four states. Descriptive statistics (frequencies) were used for characterization of the variables of interest. Residues of imazapyr (Mato Grosso and Paraná) and tricyclazole (Mato Grosso, Rio Grande do Sul, and Santa Catarina) were detected in rice samples. Glyphosate was detected in coffee (Paraná and Mato Grosso) and flour (Paraná). Glufosinate was detected in flour in the state of Santa Catarina. Glufosinate was detected in beans in all states, in addition to glyphosate (Rio Grande do Sul) and carbendazim (Mato Grosso). Although pesticide residues remained within legal limits, it should be noted that these limits do not ensure the absence of risk. Chronic exposure, especially in vulnerable populations, and the sum of diverse food sources can increase the potential for negative health effects. Exposure reduction strategies, such as opting for low-risk or organic products and applying complementary mitigation techniques, continue to be important.

## Abstract (es)

El objetivo del estudio fue analizar la presencia de residuos de plaguicidas en los alimentos básicos de la canasta básica de alimentos de los brasileños. Se recogieron muestras de azúcar refinado, arroz pulido largo fino tipo 1, café molido tradicional, harina de trigo blanca tipo 1, frijol negro común tipo 1 y aceite de soja refinado en supermercados de Mato Grosso, Paraná, Rio Grande do Sul y Santa Catarina. Los estados forman parte de la Región 3 de estratificación del Departamento Intersindical de Estadística y Estudios Socioeconómicos de la canasta básica brasileña. Los ingredientes activos analizados fueron los diez de mayor volumen comercializados para cada cultivo, de 2019 a 2023, según la información facilitada por los departamentos de agricultura de los estados. Se utilizaron estadísticas descriptivas de frecuencia para caracterizar el estudio. En muestras de arroz se detectaron residuos de imazapir (Mato Grosso y Paraná) y triciclazol (Mato Grosso, Rio Grande do Sul y Santa Catarina). En el café, se detectó glifosato en Paraná y Mato Grosso y en la harina de Paraná, mientras que se detectó glufosinato en la harina de Santa Catarina. En cuanto a los frijoles, se detectó glufosinato en todos los estados, además de glifosato (Rio Grande do Sul) y carbendazim en Mato Grosso. Aunque los residuos de plaguicidas se mantuvieron dentro de los límites legales, cabe destacar que estos límites no garantizan la ausencia de riesgo. La exposición crónica, especialmente en poblaciones vulnerables, y la suma de diversas fuentes alimentarias pueden aumentar el potencial de efectos negativos para la salud. Las estrategias de reducción de la exposición, como optar por productos de bajo riesgo u orgánicos y aplicar técnicas complementarias de mitigación, siguen siendo importantes.

## Introdução

O Brasil é um dos principais produtores e exportadores de grãos do mundo ^1^. Culturas agrícolas como os cereais, a exemplo do arroz e do trigo, e das leguminosas como, o feijão e a soja, são alimentos com apreciável valor nutricional e cultural para a população brasileira ^2^. Estes alimentos, juntamente com o café, o açúcar e outros alimentos de origem vegetal e animal, compõem o conjunto de 13 itens da Cesta Básica dos brasileiros ^3^. Antes de chegarem à mesa do brasileiro, esses alimentos percorrem etapas de produção, transporte e armazenamento, que frequentemente envolvem o uso de agrotóxicos para controle de pragas e patógenos, visando à rentabilidade da produção ^4^.

No território brasileiro, houve a comercialização de aproximadamente 755.489 toneladas de ingredientes ativos de produtos formulados em 2023 ^5^. O ingrediente ativo é o agente químico, físico ou biológico que confere eficácia aos agrotóxicos e afins ^4^. Já os resíduos são substâncias puras ou misturas remanescentes em alimentos ou no meio ambiente decorrentes do uso de agrotóxicos e afins, incluindo também os produtos de conversão e de degradação, metabólitos, produtos de reação e impurezas ^6^.

O modo de entrada dos agrotóxicos nos alimentos se caracteriza especialmente por ação sistêmica, instalando-se na matriz vegetal, e ou por contato, nas partes externas, embora uma quantidade possa ser absorvida e permanecer na matriz vegetal ^7^. É possível que os agrotóxicos, caso não tenham sido degradados pelo próprio metabolismo dos vegetais, permaneçam nos alimentos, levando os consumidores a ingerirem seus resíduos ^8^. Diante desta problemática, os resíduos nos alimentos são objeto de controle por parte de órgãos públicos de saúde ^9^.

O estabelecimento de um limite máximo de resíduo (LMR) visa assegurar que os resíduos presentes nos alimentos não constituam um risco inaceitável para a saúde dos consumidores e dos animais ^10^. Cada LMR é calculado a partir da ingestão diária aceitável (IDA). Esse parâmetro é expresso com base no peso corporal e se refere à quantidade máxima de agrotóxico que um indivíduo pode ingerir por dia, durante toda a vida, sem que sofra danos à saúde ^11^ ^,^ ^12^.

Nesse sentido, o LMR está diretamente relacionado às boas práticas agrícolas, como o cumprimento às quantidades permitidas de agrotóxicos aplicados, os ingredientes ativos autorizados para cada cultura e respeito ao período de carência ^11^. O cumprimento deste período mínimo, que decorre entre a última aplicação de um agrotóxico em uma cultura e a realização da colheita ou outra atividade, é fundamental como medida de segurança, para que o alimento não contenha resíduos de agrotóxicos acima dos limites máximos permitidos legalmente ^4^ ^,^ ^10^ ^,^ ^11^.

No Brasil, os LMRs são estabelecidos pela Agência Nacional de Vigilância Sanitária (Anvisa) ^13^. Concernente ao Mercosul, o ingrediente ativo deve ser registrado no país exportador, que deve cumprir os LMRs adotados pelo país importador e que, nos casos em que o LMR não esteja estabelecido para o produto vegetal em questão, deve-se adotar como referência, no país importador, o LMR do *Codex Alimentarius* ^14^.

Nos Estados Unidos, a Agência de Proteção Ambiental (EPA, acrônimo em inglês) realiza o monitoramento dos LMRs ^15^. Na Europa, a Comissão Europeia é responsável pelos LMRs em alimentos do bloco ^16^. As regulamentações nesse âmbito são imprescindíveis para gerar a oferta de alimentos mais seguros aos mercados nacionais e internacionais, como medidas de proteção ao meio ambiente e à saúde coletiva.

No continente europeu, a Comissão Europeia aplica um LMR padrão geral de 0,01mg/kg de alimento, quando um agrotóxico não é especificamente mencionado ^16^ ^,^ ^17^. Os limites estabelecidos pelo *Codex Alimentarius* interessam aos países exportadores, já os países importadores tendem a possuir órgãos reguladores para o controle de agrotóxicos e nem sempre adotam esses limites ^18^. Em países com legislações mais permissivas, como é o caso do Brasil, os LMRs de agrotóxicos nos alimentos tendem a ser maiores se comparados, por exemplo, aos do bloco europeu, que são mais restritivos ^19^.

Nesta lógica, em virtude da toxicidade, a segurança dos alimentos é uma preocupação dos países, uma vez que os resíduos de agrotóxicos apresentam potencial nocivo aos seres humanos. Neste cenário, a exposição aos agrotóxicos está associada a um risco carcinogênico elevado, abrangendo neoplasias hematológicas, a exemplo de leucemia, mieloma múltiplo e outras, como a neoplasia cervical ^20^ ^,^ ^21^.

O aumento na prevalência de diabetes tem sido associado à exposição aos agrotóxicos, com estudos epidemiológicos apontando para um risco elevado associado aos organoclorados e organofosforados ^22^. A exposição crônica aos agrotóxicos causa neurotoxicidade por meio de estresse oxidativo e disfunção mitocondrial, aumentando o risco para as doenças neurodegenerativas, em especial a doença de Parkinson e doença de Alzheimer ^23^ ^,^ ^24^. Da mesma forma, a exposição à essas substâncias tóxicas pode afetar negativamente a fertilidade, interrompendo a sinalização hormonal ^25^.

À luz do exposto, faz-se necessário o monitoramento de resíduos agrotóxicos nos alimentos consumidos pela população como medida de prevenção de riscos à coletividade. Neste cenário, o objetivo deste estudo foi analisar a presença de resíduos de agrotóxicos em alimentos da Cesta Básica dos brasileiros.

## Metodologia

Este estudo analisou resíduos de agrotóxicos em seis alimentos da Cesta Básica dos brasileiros. A coleta das amostras foi realizada em janeiro de 2025, em supermercados nas seguintes cidades: Sorriso (Mato Grosso), Curitiba (Paraná), Porto Alegre (Rio Grande do Sul) e Florianópolis (Santa Catarina). Os estados elencados na pesquisa fazem parte da Região 3 de estratificação do Departamento Intersindical de Estatística e Estudos Socioeconômicos (DIEESE) da Cesta Básica nacional ^3^, região em que está sediada a universidade que realizou o estudo.

As coletas foram realizadas nos supermercados de maior faturamento na capital de cada estado, conforme informado pela Associação Brasileira de Supermercados (ABRAS) ^26^. No caso do Estado do Mato Grosso, selecionou-se aleatoriamente a cidade de Sorriso, pela indisponibilidade do supermercado de maior faturamento daquele estado na capital Cuiabá.

A composição amostral englobou os seguintes alimentos em cada estado: açúcar refinado (Mato Grosso, Paraná, Rio Grande do Sul e Santa Catarian; 4 amostras de 1.000g cada), arroz polido longo fino tipo 1 (Mato Grosso, Paraná, Rio Grande do Sul e Santa Catarina; 4 amostras de 1.000g cada), café em pó tradicional (Mato Grosso e Paraná; 2 amostras de 500g cada), farinha de trigo branca tipo 1 (Paraná, Rio Grande do Sul e Santa Catarina; 3 amostras de 1.000g cada), feijão preto comum tipo 1 (Mato Grosso, Paraná, Rio Grande do Sul e Santa Catarina; 4 amostras de 1.000g cada), óleo de soja refinado (Mato Grosso, Paraná, Rio Grande do Sul e Santa Catarina; 4 amostras de 900mL cada). O café não foi coletado nos estados de Santa Catarina e Rio Grande do Sul e a farinha de trigo não foi coletada no Estado de Mato Grosso, pelo fato de não haver produção comercial das culturas nos referidos estados. Enfatizamos que o foco desta pesquisa é nos volumes de agrotóxicos comercializados em cada estado para a produção de cada alimento analisado neste estudo e não no consumo, por este motivo as amostras de café não foram coletadas no Rio Grande do Sul e Santa Catarina e de farinha de trigo no Mato Grosso.

Para a escolha e compra das marcas comerciais dos alimentos, adotou-se a marca de menor valor monetário, nos casos da disponibilidade de apenas duas marcas. No caso de três ou mais marcas disponíveis, a escolha foi pelo valor intermediário e, no caso de apenas uma marca comercial disponível, a própria marca foi considerada. Após a coleta, os alimentos foram devidamente identificados e transportados ao laboratório de análises acreditado pela Coordenação Geral de Acreditação (Cgcre) do Instituto Nacional de Metrologia, Qualidade e Tecnologia (Inmetro), conforme a norma ABNT NBR ISO/IEC 17025, sob o número CRL 0286.

Os ingredientes ativos analisados foram os dez de maior volume comercializados na produção de arroz, café, cana-de-açúcar, feijão, soja e trigo nos anos de 2019, 2020, 2021, 2022 e 2023, nos quatro estados do estudo. As informações acerca dos volumes dos ingredientes ativos comercializados em cada cultura procederam dos departamentos de agricultura dos respectivos estados (Mato Grosso: Instituto de Defesa Agropecuária de Mato Grosso - INDEA; Paraná: Agência de Defesa Agropecuária do Paraná - ADAPAR; Rio Grande do Sul: Sistema Integrado de Gestão de Agrotóxicos - SIGA; e Santa Catarina: Companhia Integrada de Desenvolvimento Agrícola de Santa Catarina - CIDASC). Salienta-se que relativo ao Estado de Santa Catarina, obteve-se acesso aos dados somente referentes aos anos de 2022 e 2023.

Após a chegada ao laboratório, as amostras dos alimentos foram analisadas seguindo metodologia validada de multirresíduos e *single* por equipamentos *gas chromatography coupled to mass spectrometry* (GC/MS - cromatografia gasosa-espectrometria de massa), *gas chromatography coupled to the detector by mass mass spectrometry* (GC/MS/MS) e *liquid chromatography coupled to mass mass spectrometry* (LC/MS/MS - cromatografia líquida-espectrometria de massa) ^27^ ^,^ ^28^ ^,^ ^29^.

Os ingredientes ativos foram analisados simultaneamente nas amostras de açúcar refinado: glifosato, diuron, tebutiurom, hexazinona, ametrina, 2,4-D, clomazona, imidacloprido, MSMA, picloram. No arroz polido longo fino tipo 1: glifosato, clomazone, imazetapir, cialofope butílico, bentazona, triciclazol, imazapique + imazapir, quincloraque, pendimetalina, florpirauxifen-benzil. No café em pó tradicional: deltametrina, glifosato, 2,4-D, ciproconazol, cletodim, clorpirifos, azoxistrobina, piraclostrobina, oxicloreto de cobre, epoxiconazol. Na farinha de trigo branca tipo 1: glifosato, 2,4-D, glufosinato sal de amônio, paraquate, propiconazol, cletodim, tebuconazol, piraclostrobina + epoxiconazol, trifloxistrobina + tebuconazol, imidacloprido. No feijão preto comum tipo 1: diquate, clorotalonil, glifosato, imidacloprido, fomesafem, glufosinato - sal de amônio, cletodim, mancozebe, tiofanato metílico, carbendazim. No óleo de soja refinado: glifosato, mancozebe, 2,4-D, acefato, clorotalonil, glufosinato - sal de amônio, diquate, paraquate, cletodim, carbendazim.

Os resultados das análises de resíduos de agrotóxicos foram apresentados por meio de relatório de declaração de conformidade emitido pelo laboratório acreditado e confrontados com os LMRs permitidos para cada alimento pela legislação brasileira ^13^. Foram utilizadas estatísticas descritivas de frequência para caracterizar o estudo.

## Resultados

Todos os ingredientes ativos analisados em todos os alimentos estavam em conformidade com a legislação brasileira ^13^. Nos casos dos alimentos (arroz, café, farinha de trigo e feijão) em que foram detectados resíduos de agrotóxicos, todos estavam abaixo do LMRs estabelecidos pela Anvisa. Em açúcar e óleo de soja não foram detectados os ingredientes ativos analisados.

Todos os resultados das análises dos ingredientes ativos nas amostras de açúcar ficaram abaixo do limite de detecção (LD), e por conseguinte também abaixo do LMR da Anvisa (Tabela 1).

**Tabela 1:** Análises de resíduos de agrotóxicos em açúcar refinado, arroz polido longo fino tipo 1, café em pó tradicional, farinha de trigo branca tipo 1, feijão preto comum tipo 1 e óleo de soja refinado, nos estados do Mato Grosso (Sorriso), Paraná (Curitiba), Rio Grande do Sul (Porto Alegre) e Santa Catarina (Florianópolis), Brasil, 2025.

Ingredientes ativos	LMR-Anvisa (mg/kg)	Mato Grosso	Paraná	Rio Grande do Sul	Santa Catarina
Açúcar refinado					
2,4-D	0,100	< 0,00067	< 0,00067	< 0,00067	< 0,00067
Ametrina	0,050	< 0,00067	< 0,00067	< 0,00067	< 0,00067
Clomazona	0,050	< 0,00067	< 0,00067	< 0,00067	< 0,00067
Diurom	0,100	< 0,00067	< 0,00067	< 0,00067	< 0,00067
Glifosato	1,000	< 0,000667	< 0,000667	< 0,000667	< 0,000667
Hexazinona	0,100	< 0,00067	< 0,00067	< 0,00067	< 0,00067
Imidacloprido	0,400	< 0,00067	< 0,00067	< 0,00067	< 0,00067
Picloram	0,020	< 0,00067	< 0,00067	< 0,00067	< 0,00067
Tebutiurom	1,000	< 0,00067	< 0,00067	< 0,00067	< 0,00067
MSMA	0,010	< 0,003333	< 0,003333	< 0,003333	< 0,003333
Arroz polido longo fino tipo 1					
Bentazona	0,100	< 0,003	< 0,003	< 0,003	< 0,003
Clomazona	0,100	< 0,003	< 0,003	< 0,003	< 0,003
Imazapique	0,050	< 0,003	< 0,003	< 0,003	< 0,003
Imazapir	0,050	0,012	< 0,010	< 0,003	< 0,003
Imazetapir	0,050	< 0,007	< 0,007	< 0,007	< 0,007
Pendimetalina	0,050	< 0,003	< 0,003	< 0,003	< 0,003
Triciclazol	8,000	0,025	< 0,003	0,021	0,032
Florpirauxifeno benzílico	0,010	< 0,003	< 0,003	< 0,003	< 0,003
Glifosato	0,200	< 0,017	< 0,017	< 0,017	< 0,017
Quincloraque	0,050	< 0,003	< 0,003	< 0,003	< 0,003
Cialofope butílico	0,010	< 0,003	< 0,003	< 0,003	< 0,003
Café em pó tradicional					
2,4-D	0,100	< 0,017	< 0,017	-	-
Azoxistrobia	0,060	< 0,003	< 0,003	-	-
Ciproconazol	0,100	< 0,003	< 0,003	-	-
Cletodim	0,500	< 0,003	< 0,003	-	-
Clorpirifos etílico	Não autorizado	< 0,003	< 0,003	-	-
Epoxiconazol	0,100	< 0,003	< 0,003	-	-
Piraclostrobina	0,500	< 0,003	< 0,003	-	-
Deltametrina	Não autorizado	< 0,003	< 0,003	-	-
Cobre	*	13,88 **	14,899 **	-	-
Glifosato	1,000	< 0,050	< 0,050	-	-
Farinha de trigo branca tipo 1					
2,4-D	0,200	-	< 0,017	< 0,017	< 0,017
Cletodim	0,500	-	< 0,003	< 0,003	< 0,003
Epoxiconazol	0,100	-	< 0,003	< 0,003	< 0,003
Imidacloprido	0,500	-	< 0,003	< 0,003	< 0,003
Piraclostrobina	1,000	-	< 0,003	< 0,003	< 0,003
Propiconazol	0,200	-	< 0,003	< 0,003	< 0,003
Tebuconazol	0,200	-	< 0,003	< 0,003	< 0,003
Trifloxistrobina	0,060	-	< 0,003	< 0,003	< 0,003
Glifosato	0,050	-	< 0,050	< 0,017	< 0,017
Glufosinato	0,500	-	< 0,017	< 0,017	< 0,050
Paraquate	Não autorizado	-	< 0,003	< 0,003	< 0,003
Feijão preto comum tipo 1					
Carbendazim	2,000	0,01	< 0,003	< 0,003	< 0,003
Cletodim	0,900	< 0,003	< 0,003	< 0,003	< 0,003
Clorotalonil	2,000	< 0,003	< 0,003	< 0,003	< 0,003
Fomesafem	0,050	< 0,007	< 0,007	< 0,007	< 0,007
Imidacloprido	0,070	< 0,003	< 0,003	< 0,003	< 0,003
Ditiocarbamatos (mancozebe)	0,600	< 0,017	< 0,017	< 0,017	< 0,017
Tiofanato metílico	0,500	< 0,003	< 0,003	< 0,003	< 0,003
Diquate	0,500	< 0,003	< 0,003	< 0,003	< 0,003
Glifosato	0,050	< 0,017	< 0,017	< 0,050	< 0,017
Glufosinato	2,000	0,058	< 0,050	< 0,050	< 0,050
Óleo de soja refinado					
Acefato	0,020	< 0,003	< 0,003	< 0,003	< 0,003
Carbendazim	0,500	< 0,003	< 0,003	< 0,003	< 0,003
Cletodim	1,000	< 0,003	< 0,003	< 0,003	< 0,003
Clorotalonil	0,500	< 0,003	< 0,003	< 0,003	< 0,003
Ditiocarbamatos	0,600	< 0,016667	< 0,016667	< 0,016667	< 0,016667
2,4-D	0,100	< 0,003	< 0,003	< 0,003	< 0,003
Diquate	0,200	< 0,0167	< 0,0167	< 0,0167	< 0,0167
Glifosato	10,000	< 0,017	< 0,017	< 0,017	< 0,017
Glufosinato	2,000	< 0,017	< 0,017	< 0,017	< 0,017
Paraquate	Não autorizado	< 0,0167	< 0,0167	< 0,0167	< 0,0167

Anvisa: Agência Nacional de Vigilância Sanitária; LMR: limite máximo de resíduo; MSMA: monossódio metanoarsenato.

* Detectado, contudo, abaixo do LMR da Anvisa;

** Metal cobre sem LMR.

Em amostras de arroz, foram detectados resíduos do ingrediente ativo imazapir no estado do Mato Grosso e Paraná. O ingrediente ativo triciclazol foi detectado em três estados (Mato Grosso, Rio Grande do Sul e Santa Catarina). Em todos os outros casos analisados, os resultados foram inferiores ao limite de detecção (LD). Salienta-se que os ingredientes ativos imazapique e imazapir foram analisados separadamente, porém compõem uma mesma formulação (Tabela 1).

O ingrediente ativo glifosato foi detectado nas amostras do café provenientes do Mato Grosso e Paraná. O metal cobre foi detectado no Mato Grosso e Paraná (Tabela 1). Todos os demais ingredientes ativos analisados apresentaram resultados abaixo do LD.

O ingrediente ativo glifosato foi detectado nas amostras de farinha no Estado do Paraná e glufosinato em Santa Catarina. Os demais ingredientes ativos apresentaram resultados abaixo do LD (Tabela 1). Os ingredientes ativos tebuconazol e trifloxistrobina foram analisados separadamente, porém fazem parte de uma mesma formulação.

O carbendazim foi detectado em amostra de feijão do Mato Grosso e o glifosato foi detectado em amostra de feijão do Rio Grande do Sul. Já o glufosinato foi detectado nas amostras de feijão de todos os estados (Tabela 1). Os demais ingredientes ativos analisados apresentaram resultados abaixo do LD.

Os ingredientes ativos analisados no óleo de soja refinado apresentaram resultado inferior ao LD em todas nas amostras (Tabela 1).

## Discussão

Este estudo investigou a presença de resíduos de agrotóxicos em alimentos que compõem aproximadamente 46% da Cesta Básica brasileira, revelando que 57,1% das amostras apresentaram resíduos detectáveis, embora todos dentro dos LMRs estabelecidos pela Anvisa ^13^. Apesar da conformidade legal, a presença de múltiplos ingredientes ativos em alimentos de consumo diário levanta preocupações quanto à segurança dos alimentos, especialmente sob a ótica da exposição crônica e dos efeitos sinérgicos entre compostos ^30^.

A literatura científica tem demonstrado que a exposição simultânea a diferentes agrotóxicos pode potencializar efeitos tóxicos, mesmo quando cada composto isoladamente está abaixo de seu LMR ^17^ ^,^ ^31^. Essa interação, conhecida como toxicidade combinatória, é particularmente relevante em alimentos como arroz, feijão e farinha de trigo, que apresentaram resíduos de dois ou mais ingredientes ativos. A ingestão repetida desses produtos da base alimentar dos brasileiros pode contribuir para bioacumulação e efeitos adversos em longo prazo, especialmente em populações vulneráveis como crianças, gestantes e idosos ^25^ ^,^ ^32^.

Embora as coletas dos alimentos tenham ocorrido no supermercado de maior faturamento em cada estado, declarado pela ABRAS ^26^, pondera-se que, em virtude do fluxo do comércio de alimentos, é possível que as amostras tenham origem de outras regiões produtoras, e não diretamente do estado de comercialização em que se realizou a coleta. Admite-se que, independentemente do estado em que o alimento foi produzido, os alimentos coletados nos pontos de venda representam a exposição real do consumidor, evidenciados pelos resultados apresentados nesse estudo.

O estudo cobriu duas das mais importantes regiões produtoras de alimentos do país, Centro-oeste e Sul, com a representatividade dos quatro estados envolvidos nessa pesquisa. A produção brasileira de arroz (*Oryza sativa* L.), café (*Coffea* sp.), cana-de-açúcar (*Sacharum* spp.), feijão preto (*Phaseolus vulgaris* L.), soja (*Glycine max* L.) e trigo (*Triticum* spp.), concentra-se nos estados do Rio Grande do Sul, Santa Catarina, Minas Gerais, Espírito Santo, São Paulo, Paraná e Mato Grosso ^1^.

As legislações dos países estabelecem LMRs distintos para cada agrotóxico nos alimentos. A Anvisa determina o LMR para o imazapir em arroz produzido no Brasil na ordem de 0,050mg/kg, enquanto, na União Europeia, sua utilização é proibida ^33^. A IDA para esse composto é de 2,5mg/kg de peso corporal ^13^. Embora seja classificado como de baixa toxicidade para seres humanos, o imazapir, quando combinado com o imazapique em formulações comerciais, apresenta ação sinérgica, ampliando sua eficácia no controle de plantas invasoras e daninhas apresentando maior efeito residual ^34^.

A comparação entre os LMRs brasileiros e os adotados por blocos econômicos como a União Europeia revela discrepâncias significativas. Por exemplo, o fungicida triciclazol, detectado em arroz neste estudo, possui LMR de 8,0mg/kg no Brasil ^13^, enquanto na União Europeia o limite é de apenas 0,01mg/kg, refletindo sua proibição naquele território ^16^. Essa diferença de 800 vezes evidencia não apenas divergências regulatórias, mas também desafios para a harmonização internacional de padrões de segurança dos alimentos. O mesmo se aplica ao herbicida glufosinato de amônio, detectado em farinha e feijão, cujo uso é proibido na Europa, mas permitido no Brasil com LMRs elevados.

O relatório de resíduos de agrotóxicos em alimentos da União Europeia de 2023 identificou inconformidades em amostras de arroz integral, majoritariamente provenientes da Índia e do Paquistão. Os resíduos acima do LMR incluíam triciclazol, propiconazol, imidacloprido e cloreto de clormequat, todos não autorizados para uso na produção de arroz dentro da União Europeia ^17^. Esse cenário evidencia não apenas a fragilidade dos controles em países exportadores, mas também a complexidade do comércio internacional de alimentos frente a legislações divergentes.

A permissividade da legislação brasileira com LMRs elevados pode comprometer a competitividade do arroz nacional em mercados mais restritivos e suscitar questionamentos sobre os critérios adotados para a definição de LMRs, especialmente diante da crescente demanda por alimentos mais seguros e sustentáveis.

O Programa de Análise de Resíduos de Agrotóxico em Alimentos (PARA) da Anvisa analisou 250 amostras de arroz produzido em território brasileiro, durante o ciclo 2023 do Plano Plurianual 2023-2025. Em 122 amostras, os agrotóxicos tebuconazol, triciclazol e pirimifos-metílico foram os que apresentaram maior número de detecções inferiores ou iguais aos LMRs, além de etiprole e glifosato e grupo dos ditiocarbamatos acima do LMR ^35^.

No presente estudo, o herbicida glifosato foi detectado em café, farinha e feijão. No café, a legislação brasileira permite um LMR de 1mg/kg, isto é, dez vezes a mais do que o permitido no café importado na União Europeia que admite 0,1mg/kg ^13^ ^,^ ^16^. Nesse cenário, o Brasil se consolida como o maior exportador de café para a Ásia, América do Norte e União Europeia, logo, essa assimetria dos LMR ultrapassa as fronteiras das legislações dos países no mercado globalizado ^36^.

Nessas condições, as exportações de alimentos do país podem enfrentar dificuldades em função de medidas sanitárias e fitossanitárias adotadas por seus parceiros comerciais. Isto tende a ocorrer pelo fato de que o Brasil apresenta, em várias situações, limites de resíduos de agrotóxicos acima daqueles adotados pelos países importadores ^18^. O PARA da Anvisa analisou, durante o ciclo de 2022, 158 amostras de café em pó. Os ingredientes ativos bifentrina, imidacloprido e azoxistrobina foram os que apresentaram maior número de detecção ^12^. Dos três ingredientes ativos, somente o fungicida azoxistrobina tem seu uso permitido na União Europeia, um importante importador do café brasileiro ^33^.

Em relação ao cobre detectado em amostras de café deste estudo, destaca-se que o fungicida e bactericida inorgânico oxicloreto de cobre foi o de maior volume comercializado para a cultura no período de estudo. Porém, devido à indisponibilidade de metodologia laboratorial para analisar o oxicloreto de cobre, o metal cobre foi analisado. A sua presença pode estar relacionada ao acúmulo no solo e captação pela planta por conta da utilização de agrotóxicos contendo cobre ou por migração por contato com equipamentos de cobre. Os limites máximos de cobre, estabelecidos como contaminantes em café em grão é de 30mg/kg, conforme a *Instrução Normativa nº 160/2022* ^37^.

No trigo produzido no Brasil, o LMR de glifosato é de 0,050mg/kg ^13^. O estudo de Costa et al. ^38^ analisou farinha de trigo adquirida no Estado de São Paulo e detectaram a presença de resíduos de bifentrina, clorpirifos, deltametrina, fenitroina e pirimifos-metilico em conformidade com os LMRs da Anvisa. O PARA da Anvisa analisou, durante o ciclo de 2022, 152 amostras de farinha de trigo. Os ingredientes ativos pirimifos-metílico, malationa e glifosato foram os que apresentaram maior número de detecção e a lambda-cialotrina e glifosato ultrapassaram os LMRs ^12^. Assim como no presente estudo, observa-se que os resíduos de agrotóxicos permanecem no alimento, mesmo após um certo grau de processamento, como é o caso da moagem do trigo em farinha.

No feijão brasileiro o LMR de glifosato é de 0,050mg/kg ^13^. O PARA da Anvisa analisou, durante o ciclo de 2022, 150 amostras de feijão e detectou 16 ingredientes ativos, dentre os 247 pesquisados. Os ingredientes ativos procimidona e carbendazim apresentaram maior número de detecção ^12^. No Estado do Paraná, foram detectados, em amostras de feijão coletados de varejo, o glufosinato de amônio, glifosato e pirimifos-metílico ^39^. O relatório de resíduos de agrotóxicos em alimentos da União Europeia de 2023 detectou, em amostras de feijões secos, o fosetil, glifosato e clorpirifós acima do LMR. Segundo este relatório, apenas o glifosato era autorizado ^17^. Estes achados são indicativos de potenciais falhas nas boas práticas agrícolas e o uso de substâncias não autorizadas para a *commodity*.

A IDA para o glifosato é de 0,5mg/kg p.c. ^13^ ^,^ ^16^. É classificado como produto improvável de causar dano agudo. Por outro lado, é declarado como disruptor endócrino e provavelmente cancerígeno para seres humanos com genotoxicidade relacionada ao ingrediente ativo e formulações (Grupo 2A) pela Agência Internacional de Pesquisa sobre o Câncer (IARC, acrônimo em inglês) ^40^.

O herbicida e regulador de crescimento glufosinato de amônio foi detectado em farinha de trigo e feijão no presente estudo. Em feijão brasileiro, o LMR de glufosinato de amônio é de 2,0mg/kg e no trigo o LMR é de 0,500mg/kg, já na União Europeia o ingrediente ativo é proibido ^13^ ^,^ ^33^. O glufosinato é considerado moderadamente tóxico para mamíferos e a IDA é de 0,02mg/kg p.c. ^13^ ^,^ ^34^. O estudo de Park et al. ^41^ avaliou que a ingestão do glufosinato é neurotóxico para seres humanos. Semelhantemente, Hsiao et al. ^32^ constataram que a exposição ao glufosinato pode causar toxicidade no sistema nervoso central e respiratório.

Além disso, a detecção de ingredientes ativos como o carbendazim, banido no Brasil desde 2022, por sua toxicidade reprodutiva e mutagênica ^42^, reforça a necessidade de vigilância contínua. A presença desse composto em amostras de feijão sugere que resíduos podem persistir mesmo após a proibição, seja por estoques remanescentes ou por contaminação cruzada.

O Brasil é um dos principais consumidores mundiais de agrotóxicos e o destino principal de múltiplos agrotóxicos proibidos na Europa ^19^. No Brasil foram comercializadas 531.483,60 toneladas dos dez ingredientes ativos de maior venda em 2023. Destes, oito eram banidos da Europa, dentre eles o acefato, atrazina, clorotalonil, dibrometo de diquate, glufosinato sal de amônio, malationa, mancozebe e S-metolacloro ^33^ ^,^ ^43^. Da lista dos dez mais vendidos no Brasil, somente os herbicidas glifosato e 2,4-D ainda são permitidos naquele bloco.

O efeito cumulativo dos resíduos de agrotóxicos consumidos sistematicamente pela população precisa ser considerado associado ao risco crônico. Nesta perspectiva, a garantia ao acesso a alimentos seguros, nutritivos e suficientes a população é um dos Objetivos de Desenvolvimento Sustentável especificamente o Objetivo 2, que discorre sobre fome zero e agricultura sustentável.

O presente estudo fornece dados substanciais relativos à detecção de resíduos de agrotóxicos em alimentos da Cesta Básica, entretanto, destacamos algumas limitações metodológicas respectivas ao delineamento analítico e amostral. Salientamos que as análises foram restritas apenas aos dez agrotóxicos mais utilizados na produção de cada alimento no período avaliado. Devido à multiplicidade e ao volume elevado de agrotóxicos empregados na produção agrícola, essa restrição analítica negligencia a potencial presença de inúmeros outros resíduos incluindo os seus metabólitos que podem estar nas amostras, mas que não foram contemplados no screening analítico.

A metodologia de amostragem do café apresentou uma restrição geográfica, excluindo o Rio Grande do Sul e Santa Catarina por não serem estados produtores desta *commodity*. O café consumido nestes dois locais provém de outros estados produtores e a exclusão de amostras pode subestimar a exposição da população que reside nesses locais, reforçando a necessidade de estudos de monitoramento focados no ponto de consumo, e não apenas na produção.

Destacamos, entre as fortalezas deste estudo, o monitoramento de resíduos de agrotóxicos em aproximadamente 46% da Cesta Básica de alimentos dos brasileiros. Os resultados desta pesquisa não apenas fundamentam a avaliação da exposição dietética dos brasileiros, mas também podem subsidiar a formulação de políticas públicas destinadas à gestão de riscos e a proteção do consumidor.

## Conclusão

Os resíduos de agrotóxicos detectados no arroz, café, farinha de trigo e feijão, encontram-se em conformidade com os LMRs permitido pela Anvisa e, segundo a legislação brasileira, estes alimentos são seguros para o consumo.

O efeito sinérgico de múltiplos agrotóxicos agindo ao mesmo tempo e a bioacumulação precisam ser considerados no universo dos riscos ao consumidor. Pondera-se que foram analisados somente os dez agrotóxicos de maior volume comercializados para cada cultura de 2019 a 2023 e que é possível haver a presença de outros resíduos não analisados, devido ao amplo uso dessas substâncias na produção agrícola de alimentos.

Recomenda-se o monitoramento dos resíduos de agrotóxicos em todos os alimentos da Cesta Básica, como ferramenta apoiada por políticas públicas de controle e vigilância em saúde da população. Salienta-se que estratégias de redução da exposição, como optar por produtos de baixo risco ou orgânicos e aplicar técnicas complementares de mitigação, continuam sendo importantes.

## Data Availability

Os dados de pesquisa estão disponíveis mediante solicitação ao autor de correspondência.
